# Low‐frequency electroacupuncture attenuates insulin resistance in a rat model of diet‐induced maternal obesity

**DOI:** 10.1002/pmf2.70312

**Published:** 2026-05-31

**Authors:** David J. Carr, Elizabeth Kelley, Morgan Sekhon, Megan Kawasaki, Keara McElroy‐Yaggy, R. Chris Skinner, Paul D. Taylor, Anna L. David, Elisabet Stener‐Victorin, Thomas L. Jetton

**Affiliations:** ^1^ Division of Maternal‐Fetal Medicine, Department of Obstetrics, Gynecology and Reproductive Sciences University of Vermont Burlington Vermont USA; ^2^ Robert Larner, M.D. College of Medicine University of Vermont Burlington Vermont USA; ^3^ Division of Diabetes, Endocrinology and Metabolism, Department of Medicine University of Vermont Burlington Vermont USA; ^4^ Department of Nutrition and Food Sciences, College of Agriculture and Life Sciences University of Vermont Burlington Vermont USA; ^5^ Department of Women and Children's Health, School of Life Course and Population Sciences Kings College London London UK; ^6^ Department of Maternal and Fetal Medicine, UCL Elizabeth Garrett Anderson Institute for Women's Health University College London London UK; ^7^ Department of Physiology and Pharmacology Karolinska Institutet Stockholm Sweden

**Keywords:** ß‐cell mass, electroacupuncture, hyperinsulinemic‐euglycemic clamp, insulin resistance, malonaldehyde, maternal obesity, placental efficiency

## Abstract

**Introduction:**

Maternal obesity is highly prevalent worldwide, conferring elevated risks of maternal/fetal complications and adult‐onset disease in offspring. Obesity and pregnancy are both states of insulin resistance. Percutaneous electrical stimulation of skeletal muscle, better known as electroacupuncture (EA), mitigates insulin resistance in polycystic ovarian syndrome and diabetes, but its efficacy in pregnancy is unknown. We aimed to determine if EA can attenuate insulin resistance in pregnant Wistar rats with high‐fat, high‐sucrose (HFS) diet‐induced obesity.

**Methods:**

Nineteen HFS‐exposed dams receiving 12–14 sessions of EA of rectus abdominis, tibialis anterior, soleus and gastrocnemius (3/15 Hz frequency, 10 mA intensity, 30 min) under isoflurane anesthesia (HFS+EA group) were compared with 19 untreated HFS‐exposed dams receiving control anesthesia (HFS group) and 10 nonobese, nonpregnant controls (Ctrl group). On embryonic day (E)19 ± 1, insulin sensitivity was measured as the steady‐state glucose infusion rate (GIR) during a gold‐standard hyperinsulinemic‐euglycemic clamp. Body composition, fasting blood glucose, fasting plasma insulin, serum corticosterone, blood pressure, pancreatic ß‐cell mass and hepatic malonaldehyde/triglyceride content were determined.

**Results:**

Compared to the Ctrl group, the HFS group exhibited a decreased GIR (mean ± SEM = 13.3 ± 1.33 vs. 18.2 ± 1.37 mg/kg/min, *p* = 0.014)—reflecting insulin resistance—as well as increased pancreatic weight (1.31 ± 0.077 vs. 1.06 ± 0.111 g, *p* = 0.04) and increased hepatic malonaldehyde content (0.24 ± 0.041 vs. 0.12 ± 0.003 µM, *p* = 0.016). These changes were reversed by EA; compared to the HFS group, the HFS+EA group had a higher GIR (16.8 ± 1.01 mg/kg/min, *p* = 0.029), lower pancreatic weight (1.03 ± 0.051 g, *p* = 0.006) and lower hepatic malonaldehyde content (0.14 ± 0.016 µM, *p* = 0.019). Visceral adiposity, insulin, corticosterone and hepatic triglyceride content were increased in the model (*p* < 0.05) but not significantly impacted by EA. There were no measurable effects of pregnancy or EA on blood pressure or ß‐cell mass, although there were significantly fewer small islets of Langerhans and significantly more large islets in HFS versus Ctrl groups. Raw fetal/placental weights did not differ; however, placental efficiency (fetal weight per unit placenta) was increased (4.09 ± 0.007 vs. 3.79 ± 0.006, *p* = 0.027) and litter size marginally decreased (median [interquartile range] = 16 [14–18] vs. 14 [11–16], *p* = 0.012) in HFS+EA versus HFS groups.

**Conclusion:**

EA attenuated insulin resistance in this rat model of maternal obesity. The effects of EA on fetoplacental growth and litter size require further investigation, especially given the latter observation could signal a potential safety issue.

## 1 INTRODUCTION

Maternal obesity is increasing in prevalence worldwide and is associated with significant maternal and neonatal morbidity, at least in part due to its associations with previously unrecognized diabetes and/or development of gestational diabetes mellitus [1]. Furthermore, offspring of obese mothers are at risk of obesity and cardiometabolic disease in adult life (via intrauterine programming effects) [2]. The rise in maternal obesity is reflective of an obesity epidemic in the general population, which has largely been driven by consumption of “Western‐style” high‐fat, high‐sucrose (HFS) diets that have a broad negative health impact, including deleterious effects on the heart and brain [3, 4]. Current approaches to the management of obesity include diet, exercise, behavioral health interventions, GLP‐1 agonists and bariatric surgery [5, 6, 7]. However, efficacy and/or access to many of these treatments are limited [8], highlighting an ongoing need to develop alternative approaches.

Obesity and pregnancy are both states of relative insulin resistance [9, 10]. Percutaneous electrical stimulation of skeletal muscle—known clinically as electroacupuncture (EA)—has been increasingly recognized as a treatment for insulin resistance outside of pregnancy. EA is an acupuncture technique that involves the insertion of fine needles into the body followed by application of a low intensity electrical current via a simulator device [11]. Glucose‐lowering effects of EA have been shown in multiple clinical and animal studies, mostly in subjects with diabetes or polycystic ovarian syndrome [12, 13, 14, 15, 16].  The neuroendocrine effects of EA are mediated by sensory afferent nerve stimulation that activates the autonomic nervous system [17] and stimulates downstream upregulation of GLUT4, MAPK and pERK in skeletal muscle [18, 19, 20]. Given increased insulin resistance in pregnancy is putatively mediated by placental secretion of insulin‐antagonizing hormones [21], it is unknown whether similar treatment effects can be achieved in the pregnant state. If so, EA may have a potential role in the prevention of gestational diabetes by enhancing insulin sensitivity in those at increased risk—for example, patients with pregestational obesity and/or polycystic ovarian syndrome. Accordingly, the aim of this study was to determine whether EA during pregnancy can attenuate insulin resistance in a rat model of maternal obesity induced by exposure to a commercially available HFS diet.

## 2 MATERIALS AND METHODS

### 2.1 Experimental design

Our initial study design comprised two groups (EA‐treated vs. untreated HFS diet‐exposed rats) and was based on an a priori sample size calculation centered on our prespecified primary outcome—steady‐state glucose infusion rate (GIR) during a hyperinsulinemic‐euglycemic clamp, which is the gold‐standard method of assessing whole‐body insulin sensitivity. Based on prior research into the effects of EA on insulin resistance in a rat model of polycystic ovarian syndrome, and assuming variability in GIR of 4 mg/kg/min (SD) and *α* = 0.05, we estimated 13 pregnant animals per group would be needed to detect a 30% increase in GIR (from 16 to 21 mg/kg/min) at 90% power [18, 22]. This target was inflated to 15 per group to account for a 10% anticipated nonresponder rate to EA. Assuming a 50% pregnancy rate [23], we procured 60 Wistar rats to generate *n* = 30 pregnancies. Ultimately, however, our required sample size was reached after batched HFS diet exposure and mating of only 50 rats, which were allocated to EA‐treated (“HFS+EA”) or untreated (“HFS”) groups (*n* = 25 each) with pregnancy rates of 76% (*n* = 19) in each. The remaining 10 rats received a normal diet and remained unmated, and thus constituted an extra (healthy nonobese, nonpregnant) control group (“Ctrl”).

### 2.2 Experimental animals

Sixty female specific pathogen‐free Wistar rats, aged 76 ± 1d and weighing 230 ± 3 g, were purchased from Charles River and housed (*n* = 3 per cage) under a 12 h/12 h light/dark cycle at 20°C–26°C and 30%–70% humidity. This work was approved by the local IACUC (#18‐066) and all procedures were carried out in accordance with the NIH *Guide for the Care and Use of Laboratory Animals* (National Academies Press) and “3Rs” principles (National Centre for the Replacement, Refinement & Reduction of Animals in Research). Fifty rats were fed ad libitum a commercial HFS diet containing 20% sucrose and providing 45% energy from fat (58V8; TestDiet) to generate a preconception state of obesity and insulin resistance. After ∼6 weeks dietary manipulation, HFS‐exposed females were co‐housed 3:1 for 72 h with male Wistar rats of equivalent age and evenly allocated based on preconception weight gain to one of two groups (*n* = 25 each): (1) the HFS+EA group that received 12–14 sessions of EA under isoflurane anesthesia between embryonic day (E)1 and E16 (where average gestation length in the Wistar rat is 21–23 days); or (2) the HFS group that underwent equivalent handling and isoflurane exposure but no EA. In both groups, pregnancy was confirmed by palpation on/after E12, and nonpregnant animals were excluded. On E18 ± 1, after a 24 h minimum “wash‐out” period to avoid acute effects of EA/anesthesia, blood pressure was measured by tail cuff using a CODA^®^ system (Kent Scientific). We included measurement of blood pressure because it has been found to be both positively and negatively impacted by EA under different experimental conditions [24, 25]. On E19 ± 1, following an 18 h overnight fast, glucose was measured in tail vein whole blood by Freestyle Lite glucometer (Abbott). Additional blood was sampled for plasma/serum generation. Finally, a terminal hyperinsulinemic‐euglycemic clamp procedure was performed, as previously described [18, 22]. The remaining 10 rats formed the Ctrl group that remained on standard chow and received neither EA nor control anesthesia. Clamps were performed at an equivalent age to those in the HFS and HFS+EA groups (and during pro‐estrus/estrus to minimize cyclic influences). All procedures and analyses were performed by investigators blinded to group allocation.

### 2.3 Electroacupuncture

Filiform, single‐use, sterile, stainless‐steel needles (diameter 0.25 mm, length 30 mm, J type; SEIRIN^®^) were inserted into rectus abdominis (T6‐L1) and bilateral tibialis anterior (L4), soleus (L5) and gastrocnemius medius (L5). The anatomic landmarks corresponded to traditional acupuncture point locations CV4, CV12, ST36, SP6 and SP9, as defined by the WHO. The innervation of these target muscles corresponds to the innervation of the pancreas, liver, mesenteric fat and uterus in the rat, and they were chosen with the experimental intent of inducing somato‐autonomic reflex effects at the appropriate spinal levels [26, 27]. Needle pairs (two needles within rectus abdominis and left/right combination for others) were stimulated with an AS Super 4 Digital EA unit (Pierenkemper) at 10 mA intensity and 3/15 Hz frequency (3s‐alternation; pulse width 210 and 120 µs for 3 and 15 Hz phases, respectively) for 30 min/day starting on E1–3 and finishing on E14–16, where E0 is the first day after overnight mating with a sperm‐positive vaginal smear and/or vaginal plug. Choice of needling location/intensity/frequency was based on prior metabolic studies of EA [12, 13] and was pragmatic (given the relative importance of each individual component of the intervention has not been clearly established). EA was administered under light isoflurane anesthesia in a mixture of 100% oxygen and air (1 L/min, 5% induction, 1%–3% maintenance).

### 2.4 Hyperinsulinemic‐euglycemic clamp

Clamp procedures were performed (at least 48 h after the final EA session) as previously described [18, 22]. In brief, deep isoflurane anesthesia was established, the rat was positioned supine on a heating mat, and the ventral neck was cleansed and incised. Sternocleidomastoid was separated in the midline to identify the left carotid artery, which was cannulated and attached to a heparinized syringe to maintain patency for serial blood sampling. The right jugular vein was identified, cannulated and attached via a three‐way cross to two high‐precision syringe pumps that were used to infuse 8 mU/kg/mL of regular insulin at a fixed rate of 0.9 mL/h, to induce mild hyperinsulinemia and suppress hepatic glucose output, followed by glucose at a variable rate. The GIR was adjusted every 5 min based on current arterial blood glucose levels until target glucose levels (108 ± 1 mg/dL) were achieved and maintained over three consecutive readings (≥15 min). A reduction in GIR reflects insulin resistance, as steady state is reached sooner when tissue responses to insulin are low and relatively more glucose remains in the circulation. Due to time constraints, an upper time limit of 2 h was set following initiation of the clamp.

### 2.5 Necropsy, tissue sampling and plasma/serum analyses

Immediately post‐clamp, rats were euthanized via thoracotomy and exsanguination by direct cardiac puncture under deep isoflurane anesthesia. Each fetus (and its placenta) was weighed and decapitated. The maternal pancreas was quickly dissected, weighed, cleaned, blotted, fixed overnight at 4°C in 4% PFA, and paraffin‐embedded. Major organs and fat depots were weighed, as well as muscles that were directly stimulated (rectus abdominis, tibialis anterior, soleus and gastrocnemius medius) or anatomically adjacent in the hind limb (gastrocnemius lateralis, extensor digitorum longus and peroneus longus). Samples of the right lateral hepatic lobe were snap‐frozen and stored at −80°C. Plasma insulin and serum corticosterone levels were measured in duplicate using rat‐specific ELISA kits #80‐CPTRT‐E‐01 (ALPCO) and #80554 (Crystal Chem). Homeostatic Model Assessment of Insulin Resistance (HOMA‐IR) indices were calculated (= [fasting insulin (µU/mL) × fasting glucose (mg/dL)] / 405). The intra‐assay coefficient of variation was <10% for both ELISAs.

### 2.6 Pancreatic and hepatic analyses

Fifteen whole pancreata (*n* = 5 per group) were sectioned into 5 µm slices that were collected at 100 µm intervals, mounted and dried overnight at 37°C–42°C (∼50 slides per pancreas). Four to five representative sections per rat (selected at 400 µm intervals to avoid double sampling of particularly large islets of Langerhans) were deparaffinized, rehydrated, rinsed, blocked, and incubated overnight with guinea pig anti‐insulin and rabbit anti‐glucagon primary antibodies, followed by donkey anti‐guinea pig CY5 and donkey anti‐rabbit CY3 secondary antibodies and DAPI. Slides were subsequently imaged on a Nikon Ti‐E morphometry workstation at 10× to allow identification of the islets (recognizable as clusters of green insulin staining surrounded by a red glucagon crown). High‐resolution images of each pancreatic section were digitally analyzed using NIS Elements software with extensive manual clean‐up. Fractional ß‐cell area was calculated by dividing the summed area of residual positive insulin staining by total pancreatic area (manually traced), averaged across the 4–5 sections per animal, and multiplied by pancreatic weight to calculate ß‐cell mass. Islet cell distribution was also examined. Finally, malondialdehyde and triglyceride content of the liver was determined using commercially available assays (#10009055 and #10010303, respectively; Cayman Chemical). Given relatively small amounts of available tissue per rat, samples within the same experimental group were homogenized and pooled for these purely exploratory analyses, which were performed in duplicate.

### 2.7 Statistical analysis

Normality of distribution was assessed using the Shapiro–Wilk test, and data were expressed as mean ± SEM or median [interquartile range (IQR)] and analyzed using the Statistical Package for the Social Sciences version 28.0. The three groups were compared using one‐way ANOVA and post hoc tests of least significant difference, or Kruskal–Wallis test, as appropriate. Pregnancy outcome data were compared between HFS and HFS+EA groups by Student's *t* or Mann–Whitney *U* tests, as appropriate. Correlations were assessed using Pearson's product moment test. Statistical significance was set at *p* < 0.05.

## 3 RESULTS

### 3.1 Body composition and whole‐body insulin sensitivity

**FIGURE 1:**
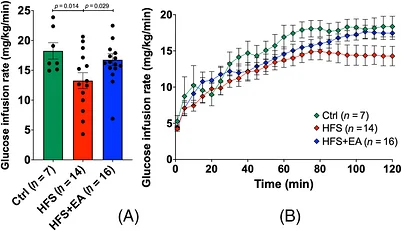
Final steady‐state glucose infusion rate (A) and temporal changes in glucose infusion rate (B) over the course of a terminal hyperinsulinemic‐euglycemic clamp procedure in 19 high‐fat, high‐sucrose (HFS) diet‐exposed dams treated with electroacupuncture (HFS+EA group), 19 untreated HFS diet‐exposed dams (HFS group) and 10 nonobese, nonpregnant controls (Ctrl group). Data are mean ± SEM. A higher glucose infusion rate indicates greater insulin sensitivity. Reduced “*n*” reflects missing date due either to technical challenges with clamp procedure (including surgical mortality and cannula leakage/disruption) or failure to achieve steady state euglycemia (defined as an arterial blood glucose level consistently 108 ± 1 mg/dL for ≥15 min) within the upper time limit set for the test (maximum 2 h).

At mating, HFS diet‐exposed rats weighed 20% more than control‐fed rats of equivalent age (327 ± 3.4 vs. 273 ± 4.4 g, *p* < 0.001) and >2 SD above genotype mean, reflecting diet‐induced obesity. The combination of diet and pregnancy induced significant insulin resistance by E18, evidenced by a decreased GIR during the clamp in HFS versus Ctrl groups (Figure 1). Insulin resistance was attenuated by EA (*p* < 0.05); accordingly, GIR did not differ between Ctrl and HFS+EA groups (*p* = 0.456). Of note, reliable primary outcome data could only be achieved in 77% of subjects due to technical challenges with the clamp procedure (surgical mortality or cannula leakage/disruption) or failure to achieve steady state euglycemia within the upper time limit set for the test (2 h), which was performed blind to group allocation. Success rates did not differ between Ctrl, HFS and HFS+EA groups (70%, 74% and 84%, respectively; *p* > 0.05). As illustrated in Figure 1A, significant variability was noted in GIR, which mirrored variability in body composition between individual animals, in keeping with our general observation that (relatively outbred) Wistar rats have a highly variable response to HFS diets, which may be attributable to genetic and/or behavioral differences. Unsurprisingly, at necropsy, HFS and HFS+EA group rats overall weighed more than Ctrl group rats (408 ± 7.1 and 388 ± 8.5 g vs. 286 ± 4.7 g; *p* < 0.001), but there was no significant difference between the two pregnant groups. Moreover, gestational weight gain did not differ between HFS and HFS+EA groups (53 ± 4.8 vs. 55 ± 3.2 g, *p* = 0.362). As shown in Table 1, all major organ and fat depot weights were increased in HFS versus Ctrl groups, while no significant differences were seen in the weights of any stimulated or anatomically adjacent muscles. Interestingly, EA negated the effect of pregnancy/diet on pancreatic weight, but had no measurable impact on other organ/depot/muscle weights.

**TABLE 1:** Necropsy data.

				*p* values			
	Ctrl group (*n* = 10)	HFS group (*n* = 19)	HFS+EA group (*n* = 19)	Overall ANOVA	Ctrl vs. HFS	Ctrl vs. HFS+EA	HFS vs. HFS+EA
Major organ weights (g)							
Liver	8.3 ± 0.501	12.2 ± 0.359	11.8 ± 0.371	**<0.001**	**<0.001**	**<0.001**	0.404
Spleen	0.59 ± 0.024	0.77 ± 0.032	0.75 ± 0.132	**0.004**	**0.002**	**0.004**	0.730
Kidney	0.99 ± 0.033	1.11 ± 0.021	1.08 ± 0.028	**0.035**	**0.011**	**0.038**	0.522
Adrenal	0.11 ± 0.005	0.14 ± 0.006	0.15 ± 0.009	**0.014**	**0.027**	**0.004**	0.333
Pancreas	1.05 ± 0.111	1.31 ± 0.077	1.03 ± 0.051	**0.015**	**0.040**	0.859	**0.006**
Internal fat depot weights (g)							
Inguinal	1.0 ± 0.10	4.1 ± 0.23	3.6 ± 0.33	**<0.001**	**<0.001**	**<0.001**	0.184
Perirenal	4.3 ± 0.31	12.8 ± 0.80	10.7 ± 0.88	**<0.001**	**<0.001**	**<0.001**	0.052
Mesenteric	3.0 ± 0.33	7.1 ± 0.64	6.3 ± 0.68	**0.001**	**<0.001**	**0.003**	0.345
Parametrial	3.7 ± 0.29	9.7 ± 0.46	8.7 ± 0.66	**<0.001**	**<0.001**	**<0.001**	0.166
Subcutaneous	4.2 ± 0.28	11.1 ± 0.86	9.6 ± 0.84	**<0.001**	**<0.001**	**<0.001**	0.175
Skeletal muscle weights (g)							
Rectus abdominis	2.26 ± 0.133	2.76 ± 0.150	2.61 ± 0.076	0.056			
Tibialis anterior	0.58 ± 0.014	0.60 ± 0.024	0.65 ± 0.026	0.112			
Soleus	0.18 ± 0.008	0.19 ± 0.009	0.20 ± 0.007	0.183			
Gastrocnemius medius	0.75 ± 0.028	0.82 ± 0.035	0.83 ± 0.020	0.172			
Gastrocnemius lateralis	0.91 ± 0.045	0.96 ± 0.039	1.05 ± 0.040	0.067			
Peroneus longus	0.18 ± 0.015	0.18 ± 0.011	0.19 ± 0.009	0.715			
Extensor digitorum longus	0.14 ± 0.009	0.15 ± 0.008	0.15 ± 0.008	0.486			

*Note*: Data are mean ± SEM. Bold text indicates *p* < 0.05.

Abbreviations: Ctrl, control; EA, electroacupuncture; HFS, high‐fat, high‐sucrose.

### 3.2 Other metabolic parameters and pancreatic ß‐cell mass

**FIGURE 2:**
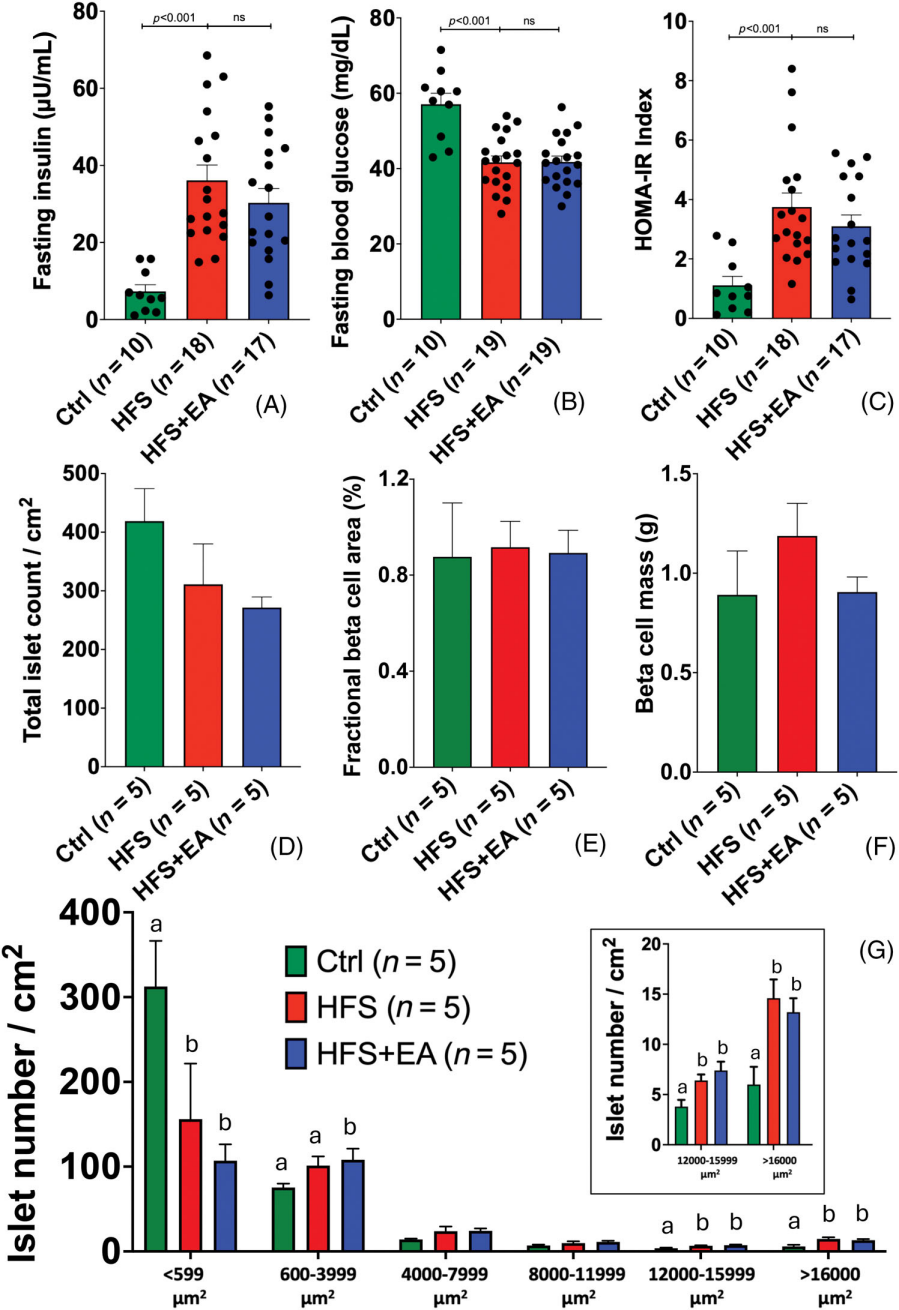
Fasting plasma insulin (A), fasting blood glucose (B) and Homeostatic Model Assessment of Insulin Resistance (HOMA‐IR) indices (C) in 19 high‐fat, high‐sucrose (HFS) diet‐exposed dams treated with electroacupuncture (HFS+EA group), 19 untreated HFS diet‐exposed dams (HFS group) and 10 nonobese, nonpregnant controls (Ctrl group). Total count of pancreatic islets of Langerhans (D), fractional ß‐cell area (E) and ß‐cell mass (F) were determined by multiple‐labeling immunofluorescence staining for insulin and glucagon followed by computerized image analysis in a subset of animals (*n* = 5 per group) and the size distribution of individual pancreatic islets of Langerhans (G) was examined. The inset shows the two smallest categories of islet sizes on an adjusted scale for clarity. Within each category, bars with different superscripts (a vs. b) are significantly different (*p* < 0.05), while bars with the same superscripts (a vs. a, b vs. b) do not significantly differ. Data are mean ± SEM.

As shown in Figure 2A,C, fasting insulin levels and HOMA‐IR indices were elevated in HFS versus Ctrl groups. Fasting glucose was lower, presumably in response to relative hyperinsulinemia of pregnancy in the setting of a prolonged fast (Figure 2B). There were no significant differences in ß‐cell fractional area, ß‐cell mass or islet counts per unit area (Figure 2D–F) but, interestingly, the HFS group exhibited significantly fewer small islets (<599 µm^2^) and significantly more large islets (>12,000 µm^2^) than the Ctrl group (Figure 2G). The group trends for ß‐cell mass notably mirrored that for pancreatic weight (Figure 2F, Table 1), which is unsurprising given it is factored into the ß‐cell mass calculation. There was a weak positive correlation between fasting plasma insulin and ß‐cell mass (*r* = 0.509, *p* = 0.026). None of these secondary metabolic outcomes were measurably impacted by EA.

### 3.3 Blood pressure, corticosterone and liver malonaldehyde/triglyceride content

**FIGURE 3:**
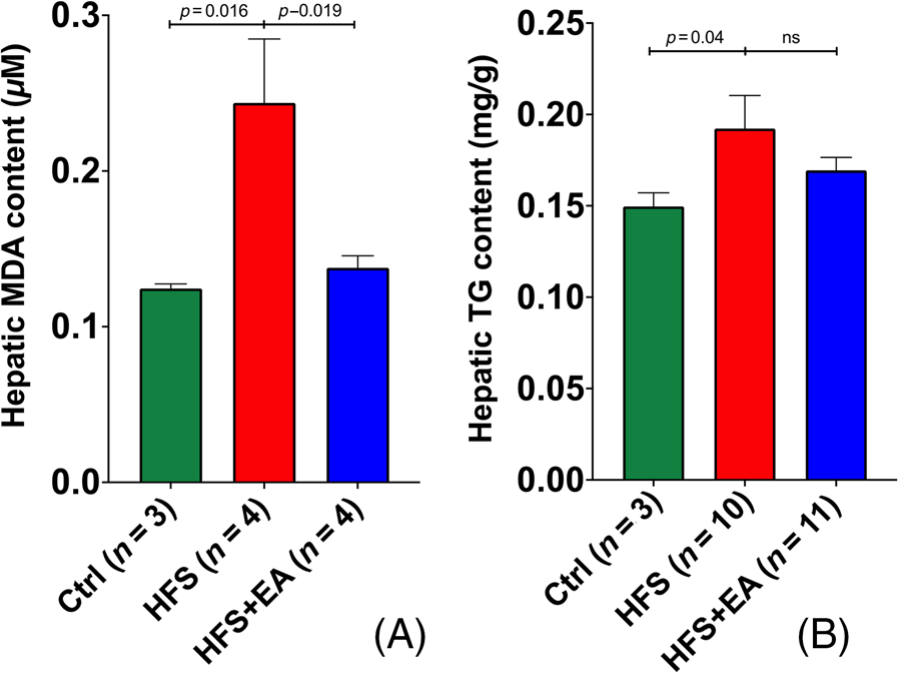
Malonaldehyde (MDA) content (A) and triglyceride (TG) content (B) of liver samples pooled from 19 high‐fat, high‐sucrose (HFS) diet‐exposed dams treated with electroacupuncture (HFS+EA group), 19 untreated HFS diet‐exposed dams (HFS group) and 10 nonobese, nonpregnant controls (Ctrl group). Data are mean ± SEM.

There were no significant differences between the Ctrl, HFS and HFS+EA groups in systolic blood pressure (145 ± 6.0, 152 ± 3.6 and 149 ± 3.3 mmHg, respectively), diastolic blood pressure (109 ± 5.3, 112 ± 3.2 and 106 ± 3.1 mmHg, respectively) or mean arterial pressure (121 ± 5.7, 124 ± 3.5 and 120 ± 3.1 mmHg, respectively). Serum corticosterone (a marker of stress) was elevated in both HFS and HFS+EA groups relative to the Ctrl group (133 ± 11.8 and 122 ± 10.5 vs. 75 ± 11.9 ng/mL; *p* = 0.004 and *p* = 0.019, respectively) but did not differ between HFS and HFS+EA groups (*p* = 0.482). As shown in Figure 3, the malondialdehyde and triglyceride content of pooled liver samples was increased in HFS versus Ctrl groups. Moreover, EA normalized hepatic malondialdehyde content.

### 3.4 Pregnancy outcome

**FIGURE 4:**
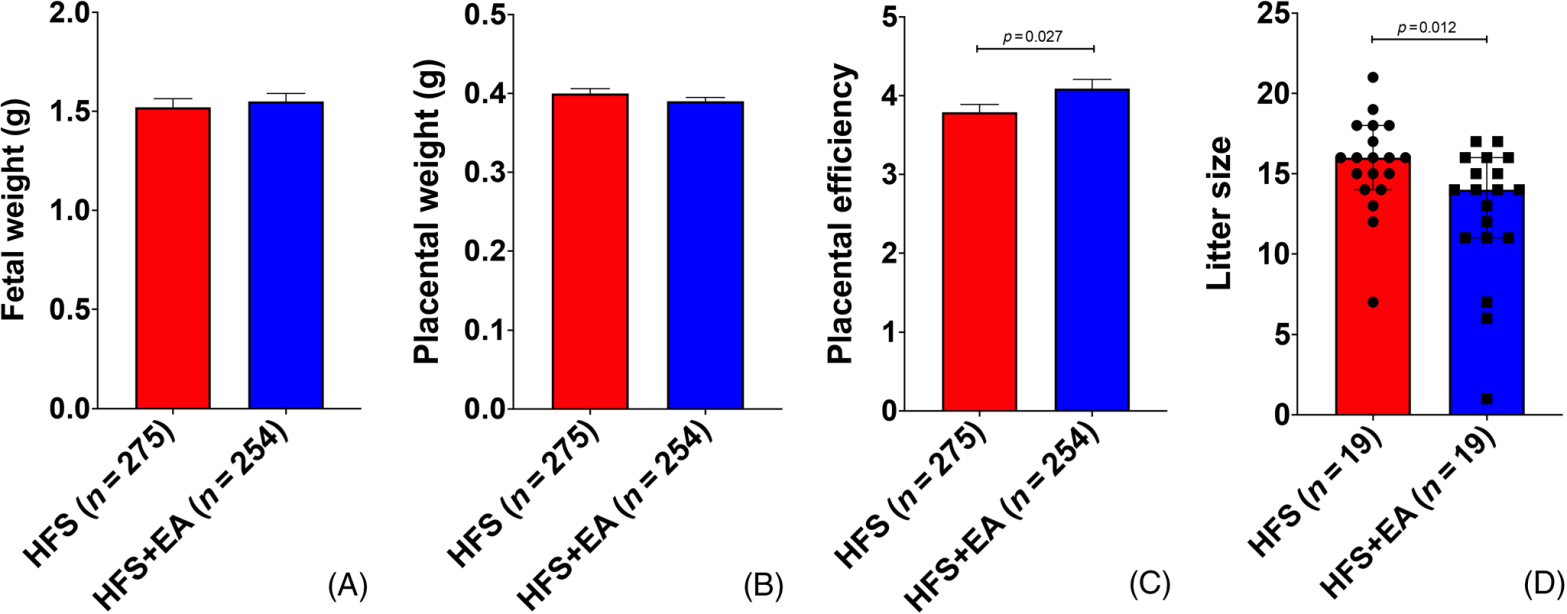
Fetal weight (A), placental weight (B) and placental efficiency (= fetal weight per unit placenta) (C) for all conceptuses of 19 high‐fat, high‐sucrose (HFS) diet‐exposed dams treated with electroacupuncture (HFS+EA group) and 19 untreated HFS diet‐exposed dams (HFS group). Data are mean ± SEM. (D) Litter size for each pregnancy in the HFS+EA and HFS groups. Data are median and interquartile range.

The raw fetal and placental weights from the 38 pregnancies did not differ between groups (Figure 4A,B); however, placental efficiency (fetal weight per unit placenta) was significantly increased by EA (Figure 4C). Median litter size was marginally but significantly lower in HFS+EA versus HFS groups (Figure 4D). Of note, reabsorptions (reflecting nonviable pups) were rare and did not differ between groups. With respect to litter size, it was notable that one pregnancy in the HFS+EA included only a single pup (a highly unusual finding in the rat), which was considered a true outlier based on Tukey's outlier test (2 × IQR below Q1). However, excluding this animal did not alter the statistical significance of the observed difference between groups (*p* = 0.012 with vs. *p* = 0.020 without), indicating this did not entirely explain the difference. Of note, when fetal weight, placental weight and placental efficiency were averaged for each individual pregnancy and compared between groups, no significant differences were demonstrated (with or without correction for the aforementioned difference in litter size).

## 4 DISCUSSION

### 4.1 Summary of main findings

In this study, we successfully established a model of maternal obesity that was characterized by a significant increase in both adiposity and whole‐body insulin resistance (evaluated using gold‐standard methodology), although the relative contributions of pregnancy versus HFS diet to these differences remain unknown. The observed increase in pancreatic weight in HFS versus Ctrl groups was presumably in compensation for increased insulin resistance (reflected in both GIR and HOMA‐IR), which is in keeping with a prior study reporting a 40% increase in pregnant Sprague–Dawley rats [28]. The most important findings of this study were that EA negated the adverse effects of pregnancy/diet on our prespecified primary outcome of whole‐body insulin resistance and prevented the increase in pancreatic weight that was observed in untreated obese dams. EA‐induced insulin sensitivity was independent of significant changes in bodyweight, adiposity, or circulating insulin or glucose in the fasting state. Of note, differences in glucose, insulin and HOMA‐IR index are difficult to interpret in the setting of such a prolonged fast during pregnancy and therefore should be considered exploratory. It is also important to note, given the lack of a healthy pregnant control group (untreated dams fed a standard rodent diet), we cannot say whether EA restored insulin sensitivity and pancreatic size to what would be expected in normal pregnancy, or overshot toward a nonpregnant baseline, and so this particular aspect requires further investigation.

### 4.2 Effects on ß‐cell mass

Of note, we demonstrated no measurable effects of pregnancy or EA on ß‐cell mass, which may either reflect confounding by dietary manipulation or lack of power, given this was an exploratory subgroup analysis (*n* = 5 per group). Accordingly, care should be taken when interpreting the ß‐cell mass results. ß‐cell mass is known to expand during pregnancy in response to physiologic insulin resistance, which is believed to occur in order to facilitate sufficient placental glucose transfer for the developing fetus, resulting in an increased demand for insulin [29]. This expansion is at least partly mediated by placental lactogens [30]. ß‐cell mass then rapidly returns to normal in the immediate postpartum period [31]. In rodents, ß‐cell mass is known to be influenced by the vagus nerve [32], the activity of which has been shown to be impacted by EA in other models [33]. Accordingly, it is feasible that EA could have exerted an effect on the pancreas via altered vagal nerve activity. Ultimately, however, we found no evidence that EA increased ß‐cell mass or circulating insulin levels. Given ß‐cell mass accounts for <1% total pancreatic weight, it is evident that changes in other pancreatic compartments must have been principally responsible for the observed differences in pancreatic weight. While relative contributions of diet/pregnancy on ß‐cell mass could not be separated herein, putative gestational increases in ß‐cell mass could be achieved through replication and hypertrophy of existing ß‐cells and/or neogenesis from various progenitors, including alpha, delta and ductal cells [34, 35]. Our observation of a relative decrease and increase in the number of the smallest and largest islets, respectively, favors enlargement of existing islets over neogenesis, potentially due to increased ß‐cell survival and/or proliferation, especially as total islet numbers were not significantly increased. A future study utilizing Ki67/bromodeoxyuridine and terminal deoxynucleotidyl transferase deoxyuridine triphosphate nick end labeling (TUNEL) staining would help delineate the relative balance between ß‐cell proliferation and apoptosis that is believed to govern temporal changes in ß‐cell mass, including those associated with pregnancy [35, 36].

### 4.3 Effects on pancreatic weight

Although glucose‐lowering and insulin‐sensitizing effects of EA have been shown in several animal models [13, 37], specific effects of EA on the pancreas have not been widely reported and, to our knowledge, no other study to date has examined the impact of EA on the pancreas in pregnant subjects. However, in nonpregnant female rats exposed to streptozotocin, high‐fat diet or both, EA attenuated abnormal islet morphology and modulated protein expression of CGRP, substance P, GLP‐1 receptor and TRPV1 in pancreatic tissues [38, 39, 40]. Most other research to date into the effects of EA on the pancreas has been in rat models of acute pancreatitis, wherein EA has been shown to: lower relative pancreatic weight; reduce pancreatic edema, necrosis and hemorrhage; enhance pancreatic expression of HSP60 and HSP72; attenuate pancreatic expression of NF‐κB; lower circulating levels of pro‐inflammatory cytokines; and partially restore impaired enteric neuronal function [41, 42, 43, 44]. A recent study examining the mechanisms of action of EA for acute pancreatitis in mice found that its effects were attenuated by vagotomy and methyllycaconitine (a selective α7 nicotinic acetylcholine receptor antagonist) [45]. This further highlights the involvement of the cholinergic anti‐inflammatory pathway, which mediates the effects of EA in rodent models of inflammation and high‐fat diet‐induced obesity [33, 46, 47, 48].

### 4.4 Potential mechanism of action

Irrespective of whether the pancreas is involved in the insulin‐sensitizing effects of EA demonstrated herein, it seems highly likely that other tissues are involved, especially considering that EA mitigated whole‐body insulin resistance without impacting circulating insulin levels, at least in the fasted state. Skeletal muscle, adipose tissue and liver are the major insulin sensitive organs [49], and all are innervated by the autonomic nervous system [50, 51, 52]. Accordingly, EA could hypothetically modulate the autonomic tone of one or more of these other tissues to increase insulin sensitivity and enhance glucose uptake. With respect to the liver, although we observed no effect of EA on hepatic weight, we found that hepatic malonaldehyde and triglyceride content was elevated in HFS versus Ctrl groups, and that the elevation in malonaldehyde was mitigated by EA. Of note, these findings were exploratory and required pooling of samples, so individual‐level analysis is required to confirm the hepatic effects of EA. While more work is required to further explore the role of the liver (including sampling the entire organ rather than a small amount), these preliminary data suggest that EA may reduce hepatic oxidative stress and lipid peroxidation, which is consistent with a prior report in healthy rats [53]. While there was no significant effect of EA on triglycerides, which are known to play a key role in insulin resistance [54], this observation warrants further investigation. With respect to skeletal muscle, EA additionally involves direct electrical stimulation (often producing rhythmic contraction) and has downstream effects similar to physical exercise [55, 56, 57, 58]. However, this is unlikely to fully account for the observed effects as, unlike exercise, the metabolic benefit of EA does not typically involve any measurable change in body weight or composition [22], and transection of somatic afferent nerves fibers to treated muscle abolished the insulin‐sensitizing effect of EA in a streptozotocin‐induced rat model of diabetes [59], favoring a neural mechanism of action.

### 4.5 Fetoplacental impact of EA

Our finding that EA increased placental efficiency is a novel and intriguing finding. Enhanced placental efficiency could indicate increased uterine blood flow, which has been shown following EA in clinical and preclinical studies [60, 61, 62, 63], and/or placental nutrient transfer. Of note, litter size was marginally lower in HFS+EA versus HFS groups and was not solely explained by the presence of an outlier in the EA‐treated group. Acknowledging this could represent a chance outcome or a potential red flag, our group is further exploring the effects of HFS diet and EA on fetoplacental growth and implantation. Additional care must be taken interpreting the placental efficiency findings given the associated differences in litter size, which could have a confounding effect. While acupuncture during pregnancy is generally considered safe [64], EA specifically in dihydrotestosterone‐exposed (but not healthy) dams was associated with higher maternal blood pressure and lower birthweight in female (but not male) pups [24]. EA did not impact blood pressure or corticosterone in this study, suggesting it did not induce acute stress in the animals. Of note, the increased corticosterone levels in HFS/HFS+EA versus Ctrl groups could represent effects of pregnancy, obesity, handling or isoflurane exposure (or any combination thereof) and warrants further exploration in future studies. It remains unknown whether this model is associated with heightened stress physiology, independent of diet. With respect to the potential fetal effects of EA (in light of enhanced placental efficiency), it will be important to determine if fetal growth symmetry, fetal glucose levels and/or placental structure are altered by EA, which will require further investigation.

### 4.6 Strengths and limitations

Relative strengths of this study include a highly controlled experimental design with blinding of investigators at all stages of the animal work, as well as tissue processing and image analysis; however, there are several limitations to be acknowledged. First, the lack of contemporaneous healthy pregnant controls (dams fed standard chow before/during pregnancy) limits our ability to differentiate effects of HFS diet versus effects of pregnancy in this model, and to identify potential interactions between these two exposures. Second, we cannot distinguish effects of high‐fat versus high‐sucrose content, which both independently impact metabolic function [65, 66]. We chose a 45%‐fat HFS diet as it better represents the “Western” human diet compared with, for example, a 60% high‐fat diet, and induces an arguably more translatable disease phenotype [66]. However, the impact of excess of each of these two major macronutrients (versus simple energy excess secondary to hyperphagia) is uncertain. Third, the duration of prepregnancy exposure to the HFS diet may have been insufficient to achieve maximal metabolic dysfunction. While a significant effect of high‐fat diets on insulin secretion is seen as early as 2 weeks, progressive effects on ß‐cell mass and islet size distribution are seen through 12–16 weeks, at least in certain rodent strains [67]. Fourth, our chosen model is characterized by insulin resistance but not hyperglycemia, which limits extrapolation of our results beyond simple maternal obesity to diabetes in pregnancy. Due to relative resistance to development of glucose intolerance, rats typically require some degree of ß‐cell injury to induce overt diabetes, which can be achieved through a high‐fat diet and streptozotocin combination [68]. Whether the insulin‐sensitizing effects of EA can be reproduced in diabetic pregnancies remains to be determined. Fifth, the use of isoflurane anesthesia introduces potential confounding of various experimental outcomes, given exposure is known to impact autonomic tone [69], insulin sensitivity [70] and stress pathways [71]. Isoflurane exposure was equivalent in HFS+EA and HFS groups, but absent in the Ctrl group, which further limits comparisons with our nonobese, nonpregnant reference group. Further work by our group is specifically evaluating the impact of isoflurane in this model. Moreover, although EA in awake rats might induce a stress response due to the nociceptive stimulus of needling and relatively high intensity of electrical stimulation employed herein, consideration is also being given to avoidance of isoflurane in the experimental groups of future studies. Sixth, with respect to the primary outcome, it must be acknowledged that missing GIR data due to clamp failure (16%–30% per group) may not be random, given these could be related to phenotype severity. However, the potential for experimental bias is arguably lessened by the correlation with effects on other parameters without missing data (e.g. pancreatic weight). Finally, the experimental design did not allow for testing of putative neuroendocrine mechanisms of action of EA in this model, which warrant further investigation.

## 5 CONCLUSION

In summary, exposure to HFS diet for ∼6 weeks preconception and throughout pregnancy generated a state of maternal obesity and insulin resistance, reflected in a lower GIR during a hyperinsulinemic‐euglycemic clamp, and this was mitigated by EA. EA also prevented the increase in pancreatic size that was seen in untreated animals relative to nonobese, nonpregnant controls. This effect was independent of a measurable change in ß‐cell mass or circulating insulin levels, therefore likely involves other metabolically active organs such as liver, skeletal muscle or adipose tissue. Finally, EA increased placental efficiency, but was associated with a marginally decreased litter size, which could represent a potential safety issue with respect to the EA intervention. This requires further investigation prior to considering clinical translation. The underlying mechanisms of action also require further exploration.

## AUTHOR CONTRIBUTIONS

David J. Carr, Paul D. Taylor, Anna L. David and Elisabet Stener‐Victorin conceived and designed the research. David J. Carr, Elizabeth Kelley, Morgan Sekhon, Megan Kawasaki, Keara McElroy‐Yaggy, R. Chris Skinner and Thomas L. Jetton performed the experiments. David J. Carr analyzed and interpreted the data, with additional interpretation by Paul D. Taylor, Anna L. David, Elisabet Stener‐Victorin and Thomas L. Jetton. David J. Carr prepared the figures and drafted the manuscript, which was edited and revised by Paul D. Taylor, Anna L. David, Elisabet Stener‐Victorin and Thomas L. Jetton. All authors approved the final version of the manuscript for submission.

## CONFLICT OF INTEREST STATEMENT

The authors declare no conflicts of interest.

## ETHICS STATEMENT

This work was approved by the University of Vermont Institutional Animal Care and Use of Committee (#18‐066). All procedures were carried out in accordance with the NIH *Guide for the Care and Use of Laboratory Animals* (National Academies Press) and “3Rs” principles (National Centre for the Replacement, Refinement & Reduction of Animals in Research).

## Data Availability

The authors agree to make the raw data from this experiment available upon request.
